# Integrating User-Centered Design and Behavioral Science to Design a Mobile Intervention for Obesity and Binge Eating: Mixed Methods Analysis

**DOI:** 10.2196/23809

**Published:** 2021-05-10

**Authors:** Andrea K Graham, Sean A Munson, Madhu Reddy, Sarah W Neubert, Emilie A Green, Angela Chang, Bonnie Spring, David C Mohr, Jennifer E Wildes

**Affiliations:** 1 Center for Behavioral Intervention Technologies Northwestern University Feinberg School of Medicine Chicago, IL United States; 2 Department of Medical Social Sciences Northwestern University Feinberg School of Medicine Chicago, IL United States; 3 Department of Human Centered Design & Engineering University of Washington Seattle, WA United States; 4 Department of Communication Studies Northwestern University Chicago, IL United States; 5 Weinberg College of Arts and Sciences Northwestern University Evanston, IL United States; 6 Department of Preventive Medicine Northwestern University Feinberg School of Medicine Chicago, IL United States; 7 Department of Psychiatry & Behavioral Neuroscience University of Chicago Chicago, IL United States

**Keywords:** obesity, binge eating, user-centered design, mobile intervention, engagement, experimental therapeutics

## Abstract

**Background:**

Accounting for how end users engage with technologies is imperative for designing an efficacious mobile behavioral intervention.

**Objective:**

This mixed methods analysis examined the translational potential of user-centered design and basic behavioral science to inform the design of a new mobile intervention for obesity and binge eating.

**Methods:**

A total of 22 adults (7/22, 32% non-Hispanic White; 8/22, 36% male) with self-reported obesity and recurrent binge eating (≥12 episodes in 3 months) who were interested in losing weight and reducing binge eating completed a prototyping design activity over 1 week. Leveraging evidence from behavioral economics on choice architecture, participants chose treatment strategies from 20 options (aligned with treatment targets composing a theoretical model of the relation between binge eating and weight) to demonstrate which strategies and treatment targets are relevant to end users. The process by which participants selected and implemented strategies and their change in outcomes were analyzed.

**Results:**

Although prompted to select one strategy, participants selected between 1 and 3 strategies, citing perceived achievability, helpfulness, or relevance as selection reasons. Over the week, all practiced a strategy at least once; 82% (18/22) struggled with implementation, and 23% (5/22) added a new strategy. Several themes emerged on successes and challenges with implementation, yielding design implications for supporting users in behavior change. In postexperiment reflections, 82% (18/22) indicated the strategy was helpful, and 86% (19/22) planned to continue use. One-week average within-subject changes in weight (–2.2 [SD –5.0] pounds) and binge eating (–1.6 [SD –1.8] episodes) indicated small clinical improvement.

**Conclusions:**

Applying user-centered design and basic behavioral science yielded design insights to incorporate personalization through user choice with guidance, which may enhance engagement with and potential efficacy of digital health interventions.

## Introduction

Experimental therapeutics and the Science of Behavior Change program at the National Institutes of Health focus on measuring whether experimentally manipulated, hypothesized targets of an intervention lead to behavior change and improved clinical outcomes [1,2]. More specifically, experimental therapeutics first evaluates an intervention effect on a hypothesized mechanism (ie, target); an intervention that engages the target mechanism is then tested to determine whether changes in the target lead to changes in clinical symptoms [3]. To date, the experimental therapeutics Research Domain Criteria framework, defined by the National Institute of Mental Health, has focused on individual-level constructs (eg, cognitive systems, positive and negative valence systems) [4]. For digital (eg, online, mobile) interventions, we have suggested that experimental therapeutics also must account for user engagement as a mediator of clinical outcomes [5] because even a clinically potent intervention will fail to improve symptoms if users do not engage with it. However, engagement is a common problem for digital interventions [6], and digital behavioral interventions have been criticized for using designs that tell users what to do, which can limit considerations for user preferences that impact engagement [7,8].

User-centered design provides a methodology for engaging deeply with end users about their needs, goals, and preferences to yield discoveries about the user experience and generate evidence for designing interventions [5,9,10]. User-centered design aims to make technologies and services engaging (eg, useful, usable, satisfying) by working collaboratively and iteratively with end users to ascertain their needs and the ways in which they interact with devices that deliver interventions [9]. As a result, digital tools achieve greater acceptability, understanding, adoption, and engagement [11-14], as well as potentially improved clinical outcomes [5,11], yet clinical scientists in health care have greatly underused design methods [15]. One reason for this underutilization may be that design methods appear to threaten the goal of maintaining fidelity to an evidence-based intervention (ie, delivering the intervention as it is intended). More specifically, conducting design activities to understand how to deliver a digital behavioral intervention could indeed result in design decisions to modify how the intervention is delivered.

However, the approach to date of simply translating an evidence-based face-to-face intervention to a digital format has not worked; the process relies on what clinicians think users need, the way in which in-person services are delivered does not align with how people engage with their phones [16,17], and it fails to take advantage of the new affordances and opportunities offered through mobile interventions [18]. Instead, new methods are needed to help our field understand how to increase engagement with digital interventions while preserving the core psychological and behavioral principles that can achieve changes in treatment targets.

**Figure 1:**
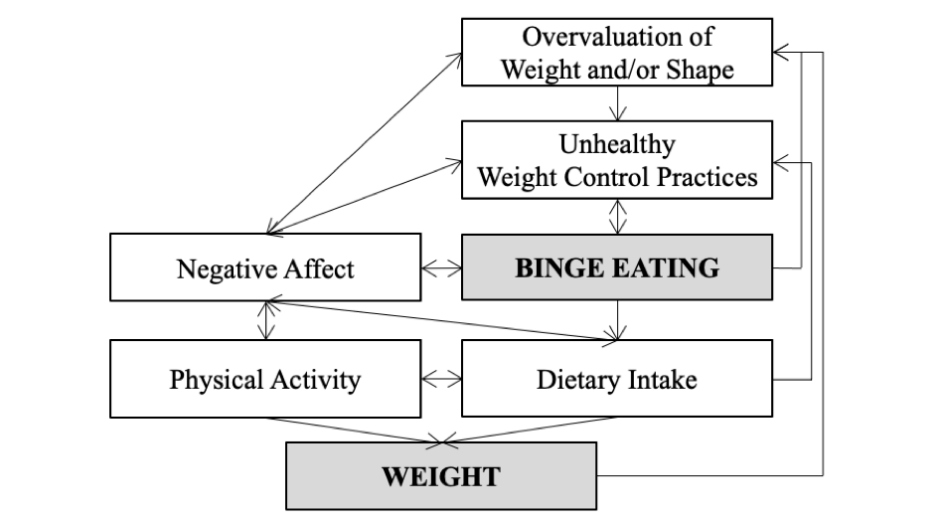
Theoretical model depicting the relation between binge eating and weight gain. The model integrates treatment targets (white boxes) in evidence-based treatments for obesity or binge eating [26-28]. Clinical outcomes are depicted in the gray boxes.

This paper aims to demonstrate the application of user-centered design and basic behavioral science to inform the design of a new mobile behavioral intervention that addresses both obesity and recurrent binge eating, an eating disorder behavior characterized by eating a large amount of food while experiencing a sense of loss of control over eating [19]. Binge eating affects up to 30% of treatment-seeking adults with obesity [20-22], and more than 75% of people with recurrent binge eating have overweight or obesity [23]. The association between binge eating and weight gain over time [24,25] makes tackling these health outcomes simultaneously important. In line with an experimental therapeutics approach [1-3], the mobile intervention design focuses on addressing putative intervention targets hypothesized as mechanisms that contribute to the cycle of binge eating and changes in weight. The theoretical model, depicted in Figure 1, integrates treatment targets in evidence-based treatments for obesity or binge eating [26-28], with the goal of delivering behavioral and cognitive strategies associated with these targets within a standalone mobile intervention. Yet intervening on these targets could be achieved through several diverse behavioral and cognitive strategies. Because of this, a design lens is needed to learn which strategies are relevant to end users and identify ways to support end users in engaging with these strategies via the planned mobile intervention.

To this end, we applied user-centered design methods to understand how end users select strategies that could address the treatment targets and how they implement these strategies over 1 week. Although this brief period may be insufficient for an end user to determine if a strategy would work for them longer term, it allowed users to answer design questions about how to make strategies within an intervention relevant and engaging without devoting substantial resources to intervention development and deployment. To avoid the shortcomings of prior, overly prescriptive digital interventions, we had users choose their strategy from multiple options. Behavioral economics suggests that leveraging choice architecture, such as using active choice (ie, forced selection among relevant options), may improve engagement and facilitate behavior change [29,30]. Accordingly, using the active choice paradigm enabled assessing if such a feature would be relevant for the mobile intervention.

Taken together, this study employed a mixed methods approach to understand how end users would select and implement a strategy and where they would struggle in the implementation process. Findings inform design implications to increase intervention engagement and, in turn, clinical impact. This work is an example of how user-centered design and basic behavioral science can be leveraged to inform the design of a mobile intervention within an overarching program of research to establish an evidence-based intervention for obesity and binge eating. Such efforts are imperative because few publications have documented the use of design methods to evaluate digital interventions for eating disorders [31], and to our knowledge, we are the first to publish on the use of design methods to create a new digital intervention for people with eating disorders [32].

## Methods

### Participants

Participants were recruited using dscout (dscout Inc), an online/mobile qualitative and market research platform. Although small for behavioral science research, this sample size is consistent with research in the field of human-computer interaction [33] and was assumed to be sufficient for achieving saturation [34-36]. This size also enabled enrolling a representative sample of target intervention users with diverse perspectives while adhering to sample size constraints in dscout.

Interested individuals were invited to complete an online screening questionnaire in dscout titled “Struggles with eating and weight” that advertised a $100 reward and 25 openings for participation and gave a brief study overview. The 15 screening questions were developed for this study to confirm eligibility; demographic data (ie, age, gender, race, city of residence) were already captured in the profile of each dscout user. Eligible participants screened positive for obesity (BMI ≥30, based on self-reported height and weight) and self-reported recurrent binge eating (≥12 episodes in the past 3 months). For reporting weight, instructions stated, “What is your current weight? Please tell us this number based on when you measure your weight wearing light indoor clothing and without shoes.” For reporting binge eating, instructions stated: “Binge eating is when someone eats an unusually large amount of food and feels a sense of loss of control while eating.” These instructions were written to align with the definition of binge eating in the *Diagnostic and Statistical Manual of Mental Disorders, Fifth Edition* [19]. Inclusion criteria required that participants were English-speaking, nonpregnant adults (aged 18 years and older), felt they weighed more than they ought to weigh (yes/no question), struggled with their weight or were interested in losing weight (yes/no question), were interested in reducing binge eating (yes/no question), and were willing to use an app to address these problems (yes/no question). Among respondents who met the criteria, the final cohort was selected to ensure diversity based on race/ethnicity, gender, and age.

### Procedure

#### Enrollment

This study was approved by the Northwestern University Institutional Review Board. All enrolled participants provided online informed consent. Of those eligible, all participants who were invited (n=25) began the study. Participation was ended early for 3 individuals who stopped completing study procedures. No reasons for discontinuation were provided. Only completers (n=22) were compensated, all of whom received the $100 compensation.

#### Dscout

All study procedures occurred online via the diary study feature of dscout [37]. Dscout has over 100,000 members who can respond to advertisements and complete screeners to determine eligibility for research opportunities. Users primarily engage with dscout via their smartphones, which facilitates capturing in-the-moment, in-context experiences over time. Multiple research prompts can be included in each diary study. Further, users can submit multiple entries to each research prompt to assess experiences across contexts. Dscout has several response formats (ie, users can upload videos that are automatically transcribed, upload pictures, submit open-ended responses, and respond to multiple-choice prompts) and has an easy-to-use interface for the researcher to interact with users as needed (eg, to send reminders).

**Figure 2:**
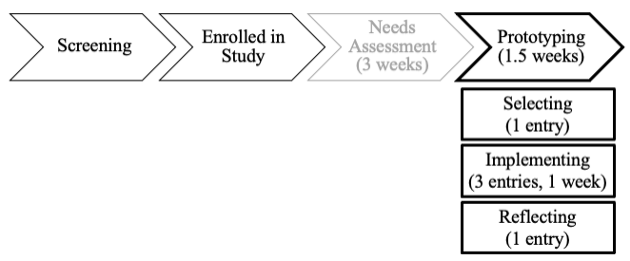
Schematic of participant flow through the study activities, including prototyping, the focus of this analysis, and the three research prompts it comprises. Of the 25 participants who enrolled, 22 began and completed prototyping.

Figure 2 presents a schematic of study activities. Participants completed design research activities over 1 month using dscout. The first 3 weeks comprised a needs assessment to learn about participants’ experiences with obesity and binge eating, strategies they have used to address these problems, and ideas for managing weight and eating. This paper focuses on the prototyping design activity that occurred in the final 1.5 weeks when participants were asked to “try making one change” to help with weight and binge eating by selecting and implementing a strategy for 1 week. Prototyping is used to iteratively evaluate design options conveyed through versions of a product (prototypes) [38]. Prototypes may or may not closely resemble the intended product, referred to as high- or low-fidelity prototypes. In this study, dscout was leveraged as a low-fidelity prototype to gain rapid insights for designing the delivery of strategies in the mobile intervention. As shown in Figure 2, the prototyping activity was administered via 3 research prompts. All 22 participants who began the prototyping activity completed it.

#### Prototyping Activity

##### Assessment Guide

An assessment guide was created that specified the research prompts that would be administered to evaluate user experiences. Questions were drafted by AKG with input from SAM, MR, DCM, and JEW. These individuals are researchers with expertise in the treatment of eating disorders and obesity, digital interventions, and/or user-centered design. AKG and SWN (an undergraduate student) then practiced answering the questions by submitting mock entries in dscout for internal testing prior to launch with participants. AKG oversaw study administration and data collection with participants. Study procedures and assessment items were consistent for all participants. After participants’ entries were submitted, SWN edited the transcribed videorecordings for accuracy and deidentification.

##### Selecting a Strategy

At the start of the week, participants were prompted to submit their first entry. In this entry, participants were asked to report their weight and number of binge eating episodes in the previous week and were then prompted to select one strategy from 20 options to practice for the week. The instructions did not indicate a limit to how many strategies participants could select. The strategies aligned with the putative intervention targets of the intervention’s theoretical model (Figure 1) and were based on evidence-based behavioral and cognitive strategies in interventions for obesity and binge eating [26-28]. Using the “think aloud” design technique [9,39], participants recorded a video while picking their strategy, talking aloud about their thought process as they made their choice. Participants then provided an open-ended response indicating their guess for how the strategy would help them.

##### Implementing the Strategy

Over the next week, participants submitted 3 entries showing moments in which they practiced or were struggling to practice their strategy. For those struggling, participants were asked to share what was getting in the way. For each entry, participants submitted a video and an open-ended response describing the experience.

##### Reflecting on Implementation

At the end of the week, participants were prompted to submit their final entry. They recorded a video reflecting on how the experience went, if it matched what they guessed would happen, and the evidence they collected on whether the strategy was helpful. Responses were coded as “planned,” “somewhat as planned,” and “not as planned.” Participants answered a yes/no question on whether they would continue using the strategy in the future and reported their weight and number of binge eating episodes over the past week.

### Analyses

Analyses focused on the process by which participants selected and implemented strategies and their change in outcomes. Qualitative analyses were conducted using Dedoose (SocioCultural Research Consultants), a qualitative data analysis software. Qualitative data from the baseline strategy selection process were coded separately from the weekly entries on implementation. Qualitative data were analyzed using thematic analysis based on the methodology of Braun and Clark [40], which involved reviewing transcripts to become familiar with the data, generating codes through open coding, iteratively applying the codes to the transcripts, and organizing the codes systematically into broader themes. AKG oversaw these analyses with input and review by SWN, SAM, and MR. Quantitative data were aggregated. The difference in weight and binge eating between the start and end of the week was calculated for each participant, the average of which was then calculated to explore average within-subject 1-week changes in weight and binge eating. Given the small sample size and exploratory nature of the quantitative analyses, a significance test was not conducted.

## Results

### Sample Characteristics

A total of 22 participants completed all study procedures and were included in the analyses. Table 1 shows demographic information on study completers. Average age was 37.0 (SD 10.2) years; 64% (14/22) identified as female. Participants identified as White (7/22, 32%), African American/Black (6/22, 27%), Hispanic/Latino (6/22, 27%), and Asian or Pacific Islander (2/22, 9%); one participant (5%) did not report their race/ethnicity. Participants reported living in 12 US states.

At screening, average BMI was 37.1 (SD 5.4, range 30.3 to 49.4), and average number of binge eating episodes over the previous 3 months was 20.5 (SD 7.3, range 12 to 35). All participants endorsed previous attempts to lose weight, and 91% (20/22) endorsed previous attempts to stop binge eating.

**Table 1:** Study participant demographics.

ID	Sex	Age	Race/ethnicity	State of residence
1	Male	42	Asian or Pacific Islander	California
3	Female	45	African American/Black	Arizona
4	Female	27	Hispanic/Latino	California
5	Female	35	Hispanic/Latino	California
6	Male	42	Hispanic/Latino	Pennsylvania
7	Male	43	African American/Black	New York
8	Female	43	Prefer not to respond	South Carolina
9	Female	45	Hispanic/Latino	Illinois
10	Female	36	White	South Carolina
11	Male	47	White	Texas
12	Female	62	White	California
13	Male	30	White	Texas
14	Female	39	Asian or Pacific Islander	Illinois
15	Female	20	White	Illinois
16	Female	43	African American/Black	California
19	Male	30	Hispanic/Latino	California
20	Female	30	White	Virginia
21	Female	22	African American/Black	Illinois
22	Male	39	Hispanic/Latino	Florida
23	Female	26	White	Ohio
24	Female	22	African American/Black	North Carolina
25	Male	45	African American/Black	New Jersey

### Selecting a Strategy

Participants selected 15 unique strategies, shown in Table 2. Although prompted to pick 1 strategy, participants selected between 1 and 3 strategies. Most participants (15/22, 68%) selected 1 strategy, 23% (5/22) of participants selected 2 strategies, and 9% (2/22) selected 3 strategies. The most commonly selected strategy was to “plan for the meals you’ll eat this week,” selected by 6 participants. The majority (25/31, 81%) of selected strategies were associated with the intervention targets of dietary intake and physical activity, whereas only 6 selections were associated with overvaluation of weight and/or shape, unhealthy weight control practices, and negative affect. The 5 strategies no one selected were associated with these latter 3 intervention targets.

**Table 2:** Selection of strategies.

Putative intervention target and strategy		Times selected	Went as planned?				Helpful^a^ %
			Yes	Some	No		
Dietary intake							
	Eat meals and snacks at the same time each day.	2	✓	✓	—^b^	100	
	Avoid eating snacks that you didn’t plan to eat.	2	✓	—	✓	50	
	Plan for the meals you’ll eat this week.	6	✓	✓✓✓✓	✓	83	
	Find a buddy who will help you eat more healthfully.	2	✓✓	—	—	100	
	Eat smaller portions.	3	✓✓✓	—	—	100	
	Eat more fruits and vegetables.	2	—	—	✓✓	50	
	Eat less fast food.	2	✓	✓	—	100	
	Total selections of this target	19	—	—	—	—	
Physical activity							
	Regularly (approximately 3 times per week) do physical activity like walking, riding a bike, or going to the gym (unless a doctor has said it is not appropriate/healthy for you to exercise right now).	4	✓✓	✓✓	—	100	
	Have less screen time: watch less television and spend less time on your computer, tablet, or phone.	1	✓	—	—	100	
	Find a buddy who will help you be more physically active.	1	—	✓	—	100	
	Total selections of this target	6	—	—	—	—	
Overvaluation of weight and/or shape							
	When you notice yourself criticizing something about your body, stop yourself. Ask yourself: What is the evidence that the criticism is true or not true? Then think of a more balanced conclusion you can draw about your body.	0	—	—	—	—	
	List things you like and value about yourself as a person. Remind yourself of things that are more important to you than how your body looks or how much you weigh.	1	✓	—	—	100	
	Avoid spending time in front of the mirror pointing out what you think of as your “flaws.”	2	✓	✓	—	100	
	Stop yourself when you dwell on “feeling fat.” Tell yourself that “fat” is not a feeling and instead say something to yourself that is not self-blaming or self-shaming.	0	—	—	—	—	
	Total selections of this target	3	—	—	—	—	
Unhealthy weight control practices							
	Avoid skipping meals or going for long stretches of time without eating.	1	—	✓	—	100	
	Avoid “dieting” and cutting out certain types of foods.	0	—	—	—	—	
	Try eating one serving of a food that you’ve been avoiding because you consider it a “trigger” food for binge eating.	1	—	✓	—	0	
	Total selections of this target	2	—	—	—	—	
Negative affect							
	Do activities that make you happy and do not involve food.	0	—	—	—	—	
	Notice times when you’re feeling down and find something that makes you feel a bit better about the situation.	0	—	—	—	—	
	Ask a friend or loved one to do something enjoyable together or repair a relationship in which you had a disagreement or falling out.	1	✓	—	—	100	
	Total selections of this target	1	—	—	—	—	

^a^Indicates the percentage of participants who rated their selected strategy as helpful to them.

^b^Not applicable.

Participants gave a variety of reasons for selecting versus not selecting strategies. Some participants indicated they selected a strategy because it was something they already were pursuing, whereas for others, this was the reason they did not select that strategy. Participants also indicated they selected strategies they perceived to be attainable and easy to complete or adjust to doing, that they perceived would be most helpful to them in managing their eating or weight, or that were new to them and therefore were perceived to be worth trying. Participants did not select strategies that they described as having been unhelpful in the past or that they believed would be unhelpful for achieving their goals around weight and binge eating. Some said certain strategies did not apply to them and thus would be challenging to achieve. Finally, participants did not select strategies because they thought they would fail in the implementation.

Participants who selected multiple strategies indicated their selections were motivated by a desire to capitalize on recent successes with those strategies or make progress with recently planned goals or because the strategies were perceived as serving a similar purpose. Two participants did not provide a rationale for why they selected multiple strategies.

While selecting strategies, all but 2 participants set a plan and/or identified ways to implement the strategy. Most participants (19/22, 86%) listed the benefits of maintaining a plan. Participants guessed their strategy would help them manage binge eating or weight (21/22, 95%), address eating-related triggers (17/22, 77%; eg, cravings, unplanned snacking, negative self-talk), or improve a related area (10/22, 45%; eg, increase self-esteem, happiness, or productivity; be more present with children; or improve their immune system).

### Implementing the Strategies

Over the week, all participants reported they practiced implementing a strategy at least once; 82% (18/22) described moments in which they struggled with implementation. A total of 23% (5/22) of participants indicated they focused on a new or additional strategy. Participants changed strategies for 2 reasons. One reason was because they realized they would be unable to work on their originally selected choice (ie, scheduling difficulties prevented exercising with a buddy). The other reason was to address a more pressing and/or relevant problem area that had presented (eg, reduce binge eating by avoiding eating unplanned snacks, increase physical activity to avoid overeating, plan for meals and snacks to reduce overeating when very hungry, or eat less fast food after a recent increase in this behavior).

Participants experienced successes and challenges with implementation; overarching themes from these results are described here, and specific details for each strategy are detailed in Multimedia Appendix 1.

The ways in which participants were successful ranged from using individually focused techniques (eg, changing a routine) to collaborating (eg, using resources and others for support); participants used techniques that best fit their needs. Participants were successful when they planned in advance, including when to eat (eg, setting a schedule), what to eat (eg, meal planning, packing healthy snacks), or how much to eat (eg, ordering smaller portions). Participants described ways they avoided triggers and unhealthy behaviors, such as avoiding eating unplanned snacks, reducing stress, or doing alternative activities to avoid overeating or triggers for overeating (eg, engaging in physical activity, referencing a list of alternative responses in the face of triggers, or using flash cards with positive statements to combat negative thoughts about their body). A total of 18% (4/22) of participants described changing their routine to engage in healthy behaviors; 18% (4/22) practiced moderation with their strategy (eg, using smaller dishes, eating smaller amounts more frequently, or eating unhealthy snacks in moderation); 23% (5/22) leveraged resources (eg, social media, commercial entities like Weight Watchers) and other people for support; 9% (2/22) found new outlets for physical activity, like being active with pets or doing chores; and 23% (5/22) described ways they challenged negative thinking, engaged in positive self-talk, and practiced motivation-enhancing techniques to support progress. During the week, 9% of participants (2/22) reflected on the results of implementing their strategy.

Participants also described challenges that affected implementation. Participants faced challenges with integrating healthy behaviors into their schedules. They had difficulties practicing their strategy in unforeseen situations (eg, when away from home or in unplanned circumstances) and challenges with sufficient planning. A total of 32% (7/22) of participants reported challenges associated with their home environment (eg, others in the home do not support healthy eating, ordering fast food is more convenient than cooking, or being at home triggered binge eating); 27% (6/22) shared how preferences for other behaviors (eg, low desire to eat healthy foods despite planning to do so or using birthdays to justify unhealthy eating) and changes in motivation affected implementation; and 14% (3/22) described the impact of stress, low mood or energy, and financial difficulties on implementation. Finally, 23% (5/22) conveyed they misunderstood the strategy and how it should be applied.

### Reflecting on Implementation

In postimplementation reflections, 41% (9/22) said implementation went as planned, 41% (9/22) said it went somewhat as planned, and 18% (4/22) said it did not go as planned. A total of 82% (18/22) endorsed their strategy as helpful. Table 2 shows these ratings relative to each strategy. Of the participants who endorsed their strategy as not helpful, 75% (3/4) said implementation did not go as planned. Finally, 86% (19/22) of participants endorsed plans to continue using their strategy.

### One-Week Changes in Weight and Binge Eating

At the start of the week, participants reported an average weight of 225.9 (SD 34.6, range 162 to 307) pounds and engaged in an average of 3.4 (SD 2.1, range 0 to 8) binge eating episodes over the prior week. After implementation, participants reported an average weight of 223.7 (SD 36.8, range 159 to 320) pounds and average of 1.7 (SD 1.2, range 1 to 5) binge eating episodes. Average within-subject changes in weight and binge eating were –2.2 (SD –5.0, range –11 to 13) pounds and –1.6 (SD –1.8, range –6 to 1) episodes, respectively.

## Discussion

### Principal Findings

User-centered design has the potential to improve engagement with and efficacy of behavioral interventions [5,9,10]. This study aimed to inform the design of a mobile intervention for obesity and binge eating by applying user-centered design and basic behavioral science to understand how end users would select and implement strategies associated with putative intervention targets for changing weight and binge eating. Results offered useful implications for intervention design and future research.

### Design Implications

In this prototyping activity, offering a choice in selecting strategies seemed to be successful given the variation of strategies participants selected. Although we did not compare active choice to no choice and therefore cannot conclude that active choice is better than assigning strategies to participants, evidence from behavioral economics shows that prompting people to make choices from several options (ie, active choice [29,30]) can spur behavior change. This may be because such an approach capitalizes on user motivation to make a change; on the whole in this study, participants selected strategies to experience success. Helping users achieve early success could make them more likely to sustain engagement; indeed, most participants endorsed plans to continue using their strategy after the week. Sustained engagement is especially important for people with obesity as failed weight loss attempts predict reduced success in future weight loss efforts [41,42]. Thus, the first design implication is to incorporate ways to offer users choices in selecting strategies and, more broadly, the areas they want to address in treatment. Offering choices also overcomes critiques of digital interventions for using designs that limit autonomy; digital interventions often have preset curricula and prescribed behavior change goals [7,8,43].

With that said, a second, complementary design implication is to reduce or scaffold the number of strategies presented. Research on decision making shows that offering too many choices leads to choice overload [44,45]. For this study, 20 options were offered. However, several entries indicated that some strategies could be collapsed, and 5 strategies were not selected. For example, no one selected “avoid ‘dieting’ and cutting out certain types of foods,” perhaps due to misunderstanding what this strategy means and why it has clinical relevance or because users may not want to follow this recommendation. A challenge for behavior change interventions is balancing what users want with what is clinically indicated when these areas may not align. It is important to incorporate designs that make less appealing but clinically necessary strategies more enticing and relevant rather than have those strategies disregarded. Designs promoting choice could be achieved through guided customization, which facilitates user choice within a defined array of options or via credible suggestions [46]. For example, it may be beneficial to deliver a distilled set of strategies [47], particularly early in the intervention, that are appealing to end users while also aligned with best clinical practices and design features that guide users to appropriate strategies and provide a rationale for their potential benefit to the user [48]. This distilled list or guided recommendations could be based on users’ identified problem areas or past progress, which could strengthen its perceived relevance to the user. Strategy selection around commonly avoided strategies also could be an area where guidance from a coach may be useful. Determining the optimal number of strategies presented at any one time remains to be tested, as does testing whether allowing users to select multiple strategies has benefits over selecting only single strategies at a time [49,50].

The third design implication is to define strategies when they are presented. Participants were not given definitions for the 20 options (to learn how users interpret the strategies) nor did they receive feedback as they shared their experiences throughout the week. As a result, seemingly straightforward strategies were interpreted in multiple ways. The variation in how participants interpreted strategies was particularly notable given that most selected strategies focused on the main intervention targets of behavioral weight loss treatment, changing dietary intake and physical activity, and 100% of participants endorsed prior weight loss attempts. This suggests that digital intervention designers cannot make assumptions about what and how much users know about eating and weight management; they need to educate users about strategies so they are positioned for success. Further, for nearly 60% of participants, implementation did not go as planned, likely because the strategies lacked specificity in how they should be implemented. Consequently, some participants abandoned their strategy or reported feelings of failure, disheartenment, and decreased willingness to practice that strategy again in the future—opposite the intention of offering choice to increase engagement. Based on these findings, an intervention architecture may need to include descriptions of what the strategy is, why the strategy is relevant to managing eating and weight, and how the strategy could be implemented. Presenting these details could help avoid misinterpretations, make unfamiliar strategies seem less daunting, and offer structure and scaffolding for their implementation.

However, delivering only instructional content on how to implement a strategy is likely insufficient. A challenge for technology-mediated services is moving users from qualitative, often distant goals to something concrete and actionable [51]. Thus, the fourth design implication is to provide support for implementing the strategy over time, something that was missing from the prototyping activity. Although results showed that participants already had some tacit understanding of evidence-based behavior change techniques (eg, planning for when, what, or how much to eat reflects action planning; avoiding eating unplanned snacks because doing so triggers overeating reflects information about antecedents) [52], many participants still struggled. Accordingly, findings suggest there would be utility in incorporating guidance and support as users implement their strategies.

Guidance and support could be delivered through coaching and content or app designs that model how to implement strategies. Throughout implementation, timely feedback on progress would be helpful, too, as this is an important component of health-related behavior change strategies [53,54] and measurement-based care more broadly [55]. Because allowing participants to implement their strategy for 1 week without feedback was problematic for some, delivering feedback soon after users begin implementation may enable users to course-correct more quickly. To balance against overly prescriptive intervention designs, corrective feedback could focus on problematic implementation (eg, when the user misunderstands the strategy). Another challenge was planning how to implement strategies, including across contexts, and executing those plans. Thus, when a strategy is presented, guidance should include designs that help users plan for implementation. At the same time, participants used existing resources to support implementation, like finding recipes and physical activity videos online. Such insights suggest it can be helpful to direct users to existing resources or help users creatively harness resources in their everyday environment, which could also save the time and costs of building app-specific versions of these resources in the intervention.

### Application of User-Centered Design to Behavioral Science

Much can be learned from this study in terms of applying user-centered design to drive progress for health-related behavioral interventions. This low-fidelity prototyping activity used qualitative and quantitative data to understand why and how users engage with aspects of an intervention—in this case, selecting and implementing strategies. The data collection platform and design methods enabled gathering in-the-moment perspectives from diverse participants who were matched to intended intervention users. End user perspectives were rapidly gathered with low participant burden, given that each entry required only a few minutes to complete and could be submitted from participants’ smartphones. Researcher burden was also minimized through the use of remote recruitment, remote data collection with multiple response types, and automatic video transcription. The design methods generated insights for intervention design without spending time or money developing a mobile intervention or creating high-fidelity prototypes. Further, these insights were gleaned from relatively few participants.

### Limitations

However, limitations should be noted. First, because procedures occurred remotely using an existing platform (ie, dscout), the research team was unable to ask clarifying or follow-up questions about participant entries, which may have limited the number and depth of insights generated. Second, the lack of definitions for each of the strategies may have influenced strategy selection and adherence and therefore generalizability and clinical relevance of the findings. Third, the study design makes it difficult to disentangle how participant improvements in the implementation process were affected by having to submit multiple entries about their progress, as longitudinal design research itself can affect behavior [56]. Also, despite asking participants to submit 3 entries showing their progress over the week, we did not ask participants to report the total number of times they implemented their strategy over the week. Fourth, although a 1-week observation period was used to gain rapid insights into strategy selection and implementation, this timeframe may have been too short for users to confidently assess the strategy’s efficacy. Fifth, the study cannot inform how users will iterate on their experiences implementing strategies or whether they will sustain engagement with implementing self-selected strategies over a longer duration. Since behavior change must occur over the long term, future design research could explore how to support users’ iterative learning over time [57]. Going forward, design recommendations should be evaluated for their impact on longer term engagement and clinical improvement. Last, although participants had small average improvements in weight and binge eating, these findings should be interpreted with caution given the brief observation period and small sample size, as achieving clinical change was not the objective of the prototyping activity. Moreover, use of self-report to assess weight and binge eating can be flawed and subject to recall biases.

### Conclusions

Results of this study highlight the translational potential of applying user-centered design and basic behavioral science to inform the design of a mobile behavioral intervention for obesity and binge eating. Discovering ways to make digital technologies relevant to end users is imperative to ensure these tools fit into the fabric of users’ lives and therefore are used in the moments and contexts when they are needed most. Such efforts can substantially improve engagement with and potential efficacy of digital health-related behavioral interventions.

## Appendix

Multimedia Appendix 1 Participant successes and challenges implementing each selected strategy.
