# Supplementary material for: Integrating User-Centered Design and Behavioral Science to Design a Mobile Intervention for Obesity and Binge Eating: Mixed Methods Analysis
Source: JMIR Form Res. 2021 May 10;5(5):e23809. doi: 10.2196/23809 (PMC8145081; doi:10.2196/23809)
Supplement: Multimedia Appendix 1 [file formative_v5i5e23809_app1.docx]

**Supplemental Table 1.** Participant successes and challenges with implementing each selected strategy.

| **Strategy (*n* who selected)** | **Successes** | **Challenges** |
| --- | --- | --- |
| **Intervention Target: Dietary Intake (7 out of 7 strategies selected)** | | |
| Eat meals and snacks at the same time each day. (*n* = 2) | Advanced planning   - - Preparing meals to have food available   Changing one’s usual routine   - - Eating earlier to prevent overeating later | Barriers with schedule   - Variable schedule impacts eating |
| Avoid eating snacks that you didn’t plan to eat. (*n* = 2) | Advanced planning   - - Plan for snacks to avoid unplanned eating   Avoiding triggers & unhealthy behaviors   - - Avoiding unplanned, available snacks   - Identifying strategies to avoid unplanned snacking   - Recognizing and avoiding triggers for unplanned snacking   Practicing moderation   - - Eating unhealthy snacks in moderation | Poor planning & unforeseen circumstances   - Unstructured meals leading to excess snacking - Not planning for a fulfilling snack   Motivation & preferences   - Celebrations “justify” unhealthy eating   Misunderstanding the strategy   - Misunderstanding about how many snacks to eat during the day |
| Plan for the meals you’ll eat this week. (*n* = 6) | Advanced planning   - - Creating a meal schedule   - Food shopping and meal prepping to have planned meals available   - Buying pre-made healthy meals   - Packing healthy snacks   Utilizing resources & others for support   - - Utilizing social media and commercial entities for ideas and motivation | Barriers with schedule   - Scheduling time for shopping or prep   Poor planning & unforeseen circumstances   - Unplanned changes to schedule   Motivation & preferences   - Low desire to eat planned foods   Emotions and energy levels   - Stress and negative affect impact motivation and planning   Finances   - Financial challenges of buying food for all planned meals   Barriers within the home environment   - Planning meals for multiple people |
| Find a buddy who will help you eat more healthfully. (*n* = 2) | Utilizing resources & others for support   - - Sharing challenges for accountability   - Having a buddy provide motivation   - Engaging friends and family for support | -- |
| Eat smaller portions. (*n* = 3) | Practicing moderation   - Eating less without avoiding all together - Using smaller dishes to manage portions - Eating smaller amounts more frequently   Cognitive restructuring & enhancing motivation   - Challenging beliefs about food | -- |
| Eat more fruits and vegetables. (*n* = 2) | Cognitive restructuring & enhancing motivation   - Willingness to try new things | Barriers within the home environment   - Lack of infrastructure to support target   Motivation & preferences   - Preference to eat other foods   Misunderstanding the strategy   - Misunderstanding the strategy to mean only eating fruits and vegetables |
| Eat less fast food. (*n* = 2) | Advanced planning   - Ordering smaller portions | Barriers within the home environment   - Inconvenience of cooking at home   Motivation & preferences   - Celebrations “justify” unhealthy eating |
| **Intervention Target: Physical Activity (3 out of 3 strategies selected)** | | |
| Regularly do physical activity (~3x/week), like walking, riding a bike, or going to the gym (unless a doctor has said it is not appropriate/healthy for you to exercise right now). (*n* = 4) | Avoiding triggers & unhealthy behaviors   - Utilizing activity to avoid overeating   Utilizing resources & others for support   - Identifying unique outlets for activity - Utilizing others to engage in activity   Changing one’s usual routine   - Changing schedule to accommodate activity   Cognitive restructuring & enhancing motivation   - Increasing motivation for self-care | Barriers with schedule   - Incorporating exercise in a busy schedule |
| Have less “screen time,” like watching less TV and spending less time on your computer, tablet, or phone. (*n* = 1) | Avoiding triggers & unhealthy behaviors   - Engaging in alternate activities   Cognitive restructuring & enhancing motivation   - Utilizing tools to sustain motivation | Misunderstanding the strategy   - Faulty understanding of unhealthy screen time |
| Find a buddy who will help you be more physically active. (*n* = 1) | -- | Barriers with schedule   - Not accounting for schedule |
| **Intervention Target: Overvaluation of Weight and/or Shape (2 out of 3 strategies selected)** | | |
| List out things you like and value about yourself as a person. Remind yourself of things that are more important to you than how your body looks or how much you weigh. (*n* = 1) | Avoiding triggers & unhealthy behaviors   - Planning and referring back to list during triggering times   Reflecting   - Reflecting on outcome | Motivation & preferences   - Avoidance due to apprehension of findings (e.g., fear of being unworthy) |
| Avoid spending time in front of the mirror pointing out things you think of as your “flaws.” (*n* = 2) | Avoiding triggers & unhealthy behaviors   - Implementing strategies to avoid triggers   Cognitive restructuring & enhancing motivation   - Focusing on positive versus negative traits - Using motivational self-talk - Avoiding perpetuating negative thoughts - Using non-judgmental/unbiased language | Poor planning & unforeseen situations   - Difficulties in unplanned situations (e.g., outside the home)   Misunderstanding the strategy   - Unrealistic expectations regarding relation between thoughts and new behaviors |
| **Intervention Target: Unhealthy Weight Control Practices (2 out of 3 strategies selected)** | | |
| Avoid skipping meals or going for long stretches of time without eating. (*n* = 1) | Advanced planning   - Planning for busy/change in schedule - Setting a plan to avoid problems with implementation | Barriers with schedule   - Not scheduling time for meals   Poor planning & unforeseen situations   - Forgetting packed meals at home   Emotions and energy levels   - Having challenges during busy/tired times |
| Try eating one serving of a food that you’ve been avoiding because you consider it a “trigger” food for binge eating. (*n* = 1) | Avoiding triggers & unhealthy behaviors   - Engaging in planned behavior and avoiding unwanted behavior | Barriers within the home environment   - Difficulty with implementation when heightened access to food |
| **Intervention Target: Negative Affect (1 out of 3 strategies selected)** | | |
| Ask a friend or loved one to do something enjoyable together. Or repair a relationship in which you had a disagreement or falling out. (*n* = 1) | Advanced planning   - Creating and implementing a plan   Avoiding triggers & unhealthy behaviors   - Reducing stress that triggers binge eating   Reflecting   - Reflecting on outcomes | -- |

**Note:** Five strategies were not selected (see Table 2 in the manuscript) and therefore are not presented in the table.

-- indicates no successes or challenges were identified in the transcripts.
